# ChatGPT's quality: Reliability and validity of concept inventory items

**DOI:** 10.3389/fpsyg.2024.1426209

**Published:** 2024-10-08

**Authors:** Stefan Küchemann, Martina Rau, Albrecht Schmidt, Jochen Kuhn

**Affiliations:** ^1^Chair of Physics Education Research, Faculty of Physics, Ludwig-Maximilians-Universität München (LMU Munich), Munich, Germany; ^2^Chair of Research on Learning and Instruction, Department of Humanities, Social and Political Sciences, ETH Zurich, Zurich, Switzerland; ^3^Chair for Human-Centered Ubiquitous Media, Institute of Informatics, Ludwig-Maximilians-Universität München (LMU Munich), Munich, Germany

**Keywords:** large language models, large foundation models, ChatGPT, physics, item creation, concept test, validation

## Abstract

**Introduction:**

The recent advances of large language models (LLMs) have opened a wide range of opportunities, but at the same time, they pose numerous challenges and questions that research needs to answer. One of the main challenges are the quality and correctness of the output of LLMs as well as the overreliance of students on the output without critically reflecting on it. This poses the question of the quality of the output of LLMs in educational tasks and what students and teachers need to consider when using LLMs for creating educational items. In this work, we focus on the quality and characteristics of conceptual items developed using ChatGPT without user-generated improvements.

**Methods:**

For this purpose, we optimized prompts and created 30 conceptual items in kinematics, which is a standard topic in high-school level physics. The items were rated by two independent experts. Those 15 items that received the highest rating were included in a conceptual survey. The dimensions were designed to align with the ones in the most commonly used concept inventory, the Force Concept Inventory (FCI). We administered the designed items together with the FCI to 172 first-year university students. The results show that ChatGPT items have a medium difficulty and discriminatory index but they overall exhibit a slightly lower average values as the FCI. Moreover, a confirmatory factor analysis confirmed a three factor model that is closely aligned with a previously suggested expert model.

**Results and discussion:**

In this way, after careful prompt engineering, thorough analysis and selection of fully automatically generated items by ChatGPT, we were able to create concept items that had only a slightly lower quality than carefully human-generated concept items. The procedures to create and select such a high-quality set of items that is fully automatically generated require large efforts and point towards cognitive demands of teachers when using LLMs to create items. Moreover, the results demonstrate that human oversight or student interviews are necessary when creating one-dimensional assessments and distractors that are closely aligned with students' difficulties.

## 1 Introduction

The development of the transformer architecture by Vaswani et al. (2017) caused a significant leap forward in natural language processing. Most importantly, the development of the generative pre-trained transformer (GPT) and the Bidirectional Encoder Representations from Transformers (BERT) model led to the widely known large language models. Most notably the release of ChatGPT caused domain-spanning technological efforts to implement it and to investigate its effectiveness. ChatGPT has been shown to exhibit benefits for several fields including medicine and education, such as scoring essays, support in diagnostic items, personalized feedback, conceptual understanding in different domains (Eysenbach et al., 2023; Kasneci et al., 2023; Steinert et al., 2023; Kieser et al., 2023; Kuroiwa et al., 2023; Kortemeyer, 2023). Despite this large range of opportunities, several authors also point toward the challenges that arise by implementing large language models (Kasneci et al., 2023; Adeshola and Adepoju, 2023; Rahman and Watanobe, 2023). In education for example, there have been concerns about misuse, ethical issues, exam fraud, incorrect outputs, as well as an overreliance by students and teachers on the output of large language models (Kasneci et al., 2023). Additionally, with the advance toward multimodality, these models became able to not only process written text but also spoken text, images, and videos, as well as to create outputs in the same formats (Küchemann et al., 2024). So, with this increasing number of opportunities they are additional challenges that arise, such as understanding and interpretation of how the output was created, including the number of used AI algorithms and unforeseen biases. Therefore, empirical research is required to examine effective ways to use these language models in education.

Previous research on large language models investigates their effectiveness in supporting prospective teachers in item development (Küchemann et al., 2023), to solve problems in physics (Krupp et al., 2024), to augment data for educational research (Kieser et al., 2023), or to provide feedback (Yin et al., 2024). However, it is not clear from these articles whether, when participants in previous studies using large language models underperformed, users had difficulty using the language model or whether the language model was generally unable to solve the item. In this context, there is a lack of quantitative analysis of the validity of the output of large language models. In this work, we analyze the validity and reliability of items that have been created with ChatGPT after careful prompt engineering. Specifically, we focus on the following research questions:

1. How are multiple-choice items created by ChatGPT rated by experts?
2. What are the item characteristics of multiple-choice items created by ChatGPT in comparison to a widely used concept test?
3. How well can ChatGPT align concept items with a previously reported factor structure of a related concept tests?

By addressing these research questions, this work will help evaluating the potential of large language models to judge the validity and reliability of the output of large language models in general, and to create assessment items for specific concept in particular.

## 2 Related work

### 2.1 Concept inventories in science

To simulate learners' conceptual understanding in science disciplines is one of the key goals of science education research. To measure conceptual understanding and to quantify the effectiveness of instructional methods, concept inventories are frequently developed and employed. Concept inventories are also a tool for formative and summative assessment, which contain ample information for students and teachers about the understanding of students and may lead to subsequent interventions (Liu, 2010). Therefore, concept inventories have a high value in science education.

According to Adams and Wieman (2011), the development of concept inventories consists of four consecutive phases. These include the delineation of the purpose of the test and the scope of the construct (phase 1), the development and evaluation of the test specifications (phase 2), the development, field testing, evaluation, and selection of the items in scoring guides and procedures (phase 3), and the assembly the evaluation of the test for operational use (phase 4). In these phases, the initially developed items are often tested with open responses first and based on the students' answers to these open questions, multiple-choice answers are formed that are closely related to students' difficulties. Moreover, the authors point toward the value of student interviews to understand the reasoning behind the answers. Therefore, the creation of concept inventories is a time-consuming task. AI technologies may help researchers at any stage during the developmental process.

Previously, in science education research, multiple concept inventories to assess students understanding have been developed, for instance, to assess the understanding of biological evolution, climate change, or Newtonian mechanics. In physics education research, the most often used concept assessment is the Force Concept Inventory (FCI) (Hestenes et al., 1992). The FCI assesses six main concepts, namely kinematics, superposition, Newton's first law, Newton's second law, Newton's third law, and kinds of forces, which are integrated in 30 items. The FCI was mainly developed by experts. Student interviews have only been used after the test development was completed. Afterwards, there have been some concerns that the concepts intended to be measured in the FCI are not confirmed in an exploratory factor analysis and that the concepts are not well reflected in students responses (Huffman and Heller, 1995; Heller and Huffman, 1995; Scott et al., 2012). Eaton and Willoughby (2018) argue that the intended factor structure not being reflected in students' responses may result from the fact that the FCI is built in an expert-like (optimal) structure and the students who answer it may also exhibit novice-like responses, which may not align well with the expert structure. In contrast, they performed a confirmatory factor analysis (CFA) testing the previously reported empirical model from the exploratory factor analysis by Scott et al. (2012) and two expert models, the original model from the FCI developers and another expert model suggested by Eaton and Willoughby (2018) with a large set of students' responses. The expert model by Eaton and Willoughby (2018) considers the fact that some of the concepts targeted in the FCI, such as Newton's second law, are not assessed in an isolated manner but rather requires also some understanding of kinematics. It consists of five factors: Newton's first law and kinematics, Newton's second law and kinematics, Newton's third law, force identification, and mixed. Using a CFA, they were able to confirm that all three models reach acceptable global fit statistics, thus describing the students' responses well (Eaton and Willoughby, 2018). In general, for an objective measure of understanding, it is necessary to isolate single attributes (concepts), i.e., to have unidimensional assessments (Planinic et al., 2019; Wright, 1997). Here, the dimensionality refers to the number of attributes of an object (here, conceptual understanding of Newtonian mechanics) being measured (Planinic et al., 2019). In our case, unidimensionality means that the items needed to be designed in the way that a certain set of items only assess a single concept and do not require the understanding of other concepts. In this way, the set of items clearly measure the understanding of this single concept. The CFA by Eaton and Willoughby (2018) showed that two of the three models that fit the FCI data do not exhibit one-dimensional factors. Therefore, an objective measure of these concepts using the FCI may be compromised.

In this work, we will build on the expert models developed earlier and study how items that assess Newtonian mechanics created using ChatGPT align with the previously reported factor models. In this way, we will see to what extent large language models are able to support researchers in the extensive effort required during the development of concept inventories and what aspects have to be considered. In the next section, we will provide an overview of the manifold potentials of large language models in education.

### 2.2 Large language model in education

Language models may exhibit a wide range of opportunities to support learners, reduce the workload of teachers, and improve the quality of teaching (see Kasneci et al., 2023 or Küchemann et al., 2024 for overviews). However, as mentioned above, there are a number of concerns, such as inaccurate output, biases, and over-reliance on the output, which might influence teachers' predisposition on using AI tools in classrooms and to support the everyday practices Regarding teachers' predisposition, Polak et al. (2022) found out that European teachers have a positive attitude toward AI for education and a high motivation to introduce AI-related content in school. According to Ayanwale et al. (2022), this is essential as the willingness of teachers to promote AI is an important prerequisite for the successful integration of AI-based technologies into the classroom. In addition, perceived usefulness, ease of use, and perceived trust in these AI-based tools are factors that need to be considered when predicting their acceptance by learners (Choi et al., 2023; Steinert et al., 2024).

However, it is not clear how large language models can support teachers in their everyday activities. For instance, Karaman and Goksu (2024) could demonstrate that ChatGPT can be used for an effective lesson planning of primary school math lessons. In comparison to students in a control group in which the teacher followed already existing lesson plans, students who took part in the lesson prepared reached a high learning gain from pre- to post-test, which was comparable to the one in the control group. Overall, the authors found that large language models are an effective tool to plan lessons. Similarly, Lee and Zhai (2024) found that ChatGPT can be effectively used for lesson planning in various subjects and that teachers reported high potentials of using ChatGPT in classroom activities.

In the context of assessment item creation, Küchemann et al. (2023) showed that prospective physics teachers can use ChatGPT to create effective physics assessments with an adequate difficulty with a high level of correctness. However, in comparison to a group of prospective physics teachers who used a textbook to create physics items, the ones who used ChatGPT struggle to integrate the items in a meaningful context and the items exhibited a lower clarity. In addition, both groups had difficulties to provide all necessary information that are relevant to solve items. Similar to this work, several other works offer a qualitative analysis of ChatGPT's output and draw conclusions on potential applications of ChatGPT in education (see for instance Ausat et al., 2023; Krupp et al., 2024, 2023).

However, in case of an insufficient performance of large language models, it is often unclear whether the users lack sufficient proficiency in using large language models or the language model itself is incapable of providing appropriate assistance. In this work, we analyze the quality of ChatGPT's output after refined prompt engineering without manually modifying the outputs. We chose the specific case of concept item creation, as it is a regularly re-occurring activity in teacher practices. In this way, we are able to provide insights into the quality of ChatGPT's output and into what teachers need to consider when using large language models for item creation.

## 3 Methodology

### 3.1 Participants

In total, 209 undergraduate STEM students from the University of Wisconsin (UW), Madison, and 51 undergraduate physics students from the Ludwig-Maximilians-University (LMU) München took part in the study. The participation was rewarded with 10 EUR at LMU and a 20 USD-Amazon gift card at UW-Madison. We excluded students who did not complete the study or who answered one of the control questions incorrectly (see below). In addition to the control questions, we carefully evaluated the time invested in answering the test items to account for the fact that some students may simply skip through the test items without thinking about the answers. We found that there was one student who reached a rather high score of 82% in a time of 14 min and 44 sec. The scores of students who completed the questionnaire below this time are fluctuating around the probability of guessing, which likely indicates that they guessed the answers. Therefore, we set a time of 14 min and 44 sec as threshold for including students' answers in the analysis. The students who completed the questionnaire below this time threshold were excluded from the analysis. These exclusion criteria led to a final data set of *N*= 173 students (*N*= 67 female, *N*= 91 male, *N*= 3 other, and *N*= 12 made no statement, average age *M*= 20.6 years) who were considered in the analysis.

In this work, students covered the topics assessed in the FCI and the GPT items, namely the concepts of motion and Newton's mechanics, at least one time prior to this study. In Germany, the physics curricula in schools of every state cover the concepts of motion and Newton's mechanics, and they are a part of Disciplinary Core Ideas in Wisconsin's Standards for Science. Moreover, understanding motion and forces is also part of the National Science Education Standards and part of the Next Generation Science Standards. Therefore, it is reasonable to assume that, the students had covered the concepts covered by the FCI and the GPT items.

### 3.2 Item creation

We chose kinematics and forces as the topics of the items as they are widely covered in school curricula across countries, and intensively researched topics in physics education research. Additionally, the FCI is the most used concept inventory in physics education, and its factor structure is well known.

**Figure 1:**
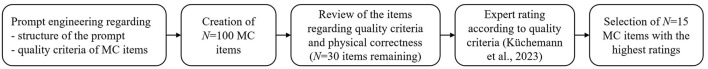
Systematic creation of kinematic items using ChatGPT 3.5.

ChatGPT 3.5 was prompted to create items that target five subcategories of the FCI: kinematics (i.e., velocity and acceleration), Newton's first law, Newton's second law, Newton's third law, and the superposition. We applied a systematic sequence of items design using on ChatGPT consisting of the following five steps (Figure 1).

**Figure 2:**
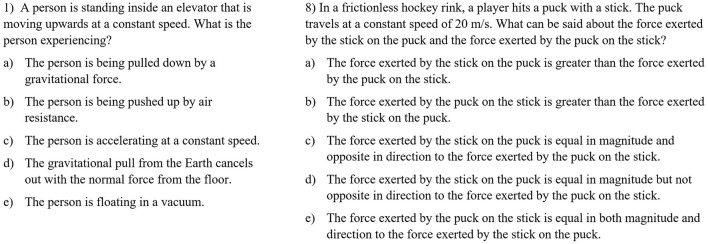
Two example items to assess Newton's 1st law (1) and Newton's 3rd law (8) created by ChatGPT.

1. We developed a prompt that includes the following characteristics of the items: multiple-choice (MC) items with five answer alternatives including one correct answer, the items should be embedded in a reasonable context, they should not contain images and only consist of a written text, and the item should not mention the physical principle required to solve the item. We mention these characteristics in the prompts as we saw that they made a difference. In contrast, additional specifications such as “design the items for high school level or introductory university level physics” or “include a cognitive activity of ‘applying' according to Bloom's taxonomy” did not cause a notable difference to the created items.
2. We used this prompt to create *N* = 100 multiple choice items, *N* = 20 for each of the five categories.
3. During the initial selection process, 70 items were eliminated, which led to a set of *N* = 30 items. The reasons for eliminating these items were:

- The context of the item describes a physically unrealistic or incomplete scenario (Q: “If an object is at rest, what force is acting upon it to keep it at rest?”)
- The item was identical or very similar to another item.
- It was obvious that the answer alternatives of the item were all incorrect or that multiple answers were correct or a correct answer was stated as incorrect (Question: Which of the following descriptions of the behavior of a falling stone is most accurate? Presumable incorrect answer: “A stone will fall faster and faster as it approaches the ground”).
- The presumable correct answer alternative did not fit to the context described in the item (Question: “Which of the following descriptions of the behavior of a falling stone is most accurate?” Presumable correct answer: “The speed of the stone remains the same unless an external force acts on it”).
- At least one of the answer alternatives were physically incorrect (Answer alternative: “The stone will stop falling after it has been lying on the ground for some time.”)
- The item stem contained the concept that needs to be applied to solve the item (example: “Which of the following best describes Newton's first law in the context of a stone falling to the ground?”)
- The item stem already contained the correct answer (Q: “Imagine you are driving a car on a straight road at a constant speed. What can be said about the motion of the car?”; A: “It moves at a constant speed in a straight line.”)

4. Next, we performed an expert rating based on 15 quality criteria with two experts from physics education research to ensure the content validity of the GPT items. Seven of these criteria have been reported earlier by Küchemann et al. (2023). Eight additional criteria were used to account for the specific question type (MC questions) and that they have been exclusively developed by ChatGPT without human revision. The criteria and ratings are shown in the Supplementary material. The rating resulted in an interrater reliability in terms of Cohen's kappa κ = 0.4, which means that there was a moderate agreement between the two raters. The conflicts between the two raters were adjudicated after discussion.
5. In the expert ratings of the items, each of the criteria could be either rated with 1 (in case it applies to an item) or 0 (in case does not apply to an item) points. Adding up these points of all criteria for a single item lead to a final “score” of each item. We selected three items with the highest final score in each of the five subcategories, Newton's 1st Law, Newton's 2nd law, Newton's 3rd law, kinematics and superposition. This lead to a final set of 15 items. The final set of GPT items contained only 15 items, because we did not want to have too many items to avoid students from filling out the questionnaire incompletely. The average final scores of the selected items in each category are: Newton's 1st Law: 0.98, Newton's 2nd law: 1.00, Newton's 3rd law: 0.98, kinematics: 0.98, and superposition: 0.73. This means that the items in categories Newton's 1st Law, Newton's 2nd law, Newton's 3rd law, kinematics reached an excellent expert rating, but the items in the context of superposition reached only a moderate score. Two example items for the concept Newton's 1st Law and Newton's 3rd law are shown in Figure 2.

During the entire process, we did not manually modify or revise the items created by ChatGPT.

### 3.3 Expert ratings of ChatGPT items

The expert rating can be found in the supporting online material. Regarding the item ratings, the experts rated nine out of 15 items with the highest score. Three of the remaining six items reached a relative score of 0.93, which means that experts only considered that one of the 15 criteria was not fulfilled. Specifically, the experts considered each of these three item stems as misleading. The remaining three items were all in the category of superposition, and they were rated with a score between 0.67 and 0.80. In two of these three items, the item stem was rated as misleading and insufficiently specified.

Regarding specific criteria, the answer alternatives of five of the fifteen selected ChatGPT items and the item stem of two items were rated as misleading. Furthermore, the item stem of two of the ChatGPT items were rated to have an insufficient specificity (in line with previous findings by Küchemann et al., 2023), and in two items the answer alternatives were rated as ambiguous.

However, all selected ChatGPT items were rated as scientifically correct, the answer alternatives were not too similar, they contained one correct and four incorrect answer alternatives, were relevant to assess the target concept, had an adequate difficulty, targeted a single concept, required a cognitive activity that was related to Bloom's taxonomy levels “apply” and “evaluate,” and they were embedded in an appropriate context.

In sum, this means that experts thought that all selected items created by ChatGPT fulfilled important quality standards, but ChatGPT sometimes created items with misleading item stem and answer alternatives, especially for specific concepts such as superposition. The overall lower ratings of items related to the concept of superposition suggests that ChatGPT has difficulties with targeting specific concepts.

### 3.4 Administration

The items created by ChatGPT (15 items) were administered together with the FCI items (the 30 items) in randomized order as an online survey. Additionally, we added 5 simple control questions (such as “what color is the sky?”), evenly distributed among the other items. If a student did not answer one of these control questions correctly, all answers of the student would not be considered in the analysis.

## 4 Results

### 4.1 Classical test theory

**Figure 3:**
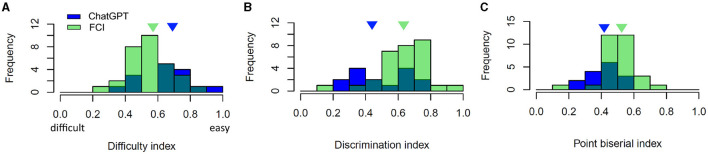
Item characteristics of MC items of the FCI (green) and those created with ChatGPT (blue): **(A)** Difficulty index, **(B)** Discrimination index, and **(C)** Point biserial index. The triangles indicate the median of ChatGPT items (blue) and FCI items (green).

We evaluated the difficulty index, discriminatory index and point-biserial index according to the classical test theory (Figure 3), (Ding and Beichner, 2009).

Here, the difficulty index was determined by


(1) $$\begin{array}{l} P=\frac{N_{1}}{N}, \end{array}$$

where *N*_1_ denotes the number of students who correctly solved the item and *N* is the total number of students.

For the determination of the discrimination index, the students are divided into quarters based on their performance in the whole item set. The 25% of the highest scoring students are in the *H*-group and the 25% lowest scoring students are in the *L*-group. Now, we determined the number of correct responses in the respective item by the *H*-group (*N*_*H*_) and by the *L*-group (*N*_*L*_). The discrimination index can then be determined by


(2) $$\begin{array}{l} D=\frac{N_{H}-N_{L}}{N/4}, \end{array}$$

where *N* indicates the total number of students.

The point-biserial index (or item-test correlation) is determined by


(3) $$\begin{array}{l} r_{pbs}=\frac{{\bar{X}}_{1}-\bar{X}}{\sigma_{X}}\sqrt{\frac{P}{1-P}}, \end{array}$$

where ${\bar{X}}_{1}$ is the average total score of students who correctly solved the respective item, *X* is the average total score of all students, σ_*X*_ is the standard deviation of the total score of the entire sample, and *P* is the difficulty index for the respective item.

*Difficulty index:* In Figure 3, we can see that the difficulty index of GPT items ranges from 0.37 to 0.91. Therefore, the GPT items covers the entire suggested range of difficulty of 0.3 to 0.9 according to Ding and Beichner (2009). Here, a low difficulty index means that the item was difficult and a high value means that the item was easy for students. The median of the difficulty index of GPT items is 0.69, and the one of FCI items is 0.57, i.e., the GPT items were easier for students.

*Discrimination index:* Overall, we found that the median discrimination index of GPT items is 0.45, which is in a satisfactory range >0.3 according to Ding and Beichner (2009). Two items fall below this threshold. These two items have a high item difficulty index and therefore are prone to insufficiently discriminate between good and poor performers. The median discrimination index of the FCI is 0.62. This implies that the FCI is better able to discriminate between good and poor performers.

*Point-biserial index (Item-test correlation)*: The item-test correlation coefficients of all items are above the expected level of 0.2. This indicates good item consistency and that each item is consistent with the other items in the test (Ding and Beichner, 2009). The median of the point-biserial index of the GPT items is 0.43, whereas the median point-biserial index of the FCI is 0.52. This means the GPT items have a slightly lower consistency than the FCI. It is in agreement with the following analysis of reliability.

*Reliability:* To measure the internal reliability of the ChatGPT items, we determined Cronbach's α = 0.74. The result of Cronbach's α is expected to be lower than the one of the FCI items because of the smaller number of ChatGPT items (*N* = 15) in comparison to the number of FCI items (*N* = 30). To account for this difference, we estimated the value of Cronbach's α for a hypothetical set of *N* = 30 ChatGPT items using the Spearman-Brown formula. This leads to an estimated value of Cronbach's α = 0.85. In comparison, the reliability of the FCI items is α = 0.90. This means that the GPT items reached a good reliability, whereas the FCI exhibit an excellent reliability.

### 4.2 Convergent validity

**Figure 4:**
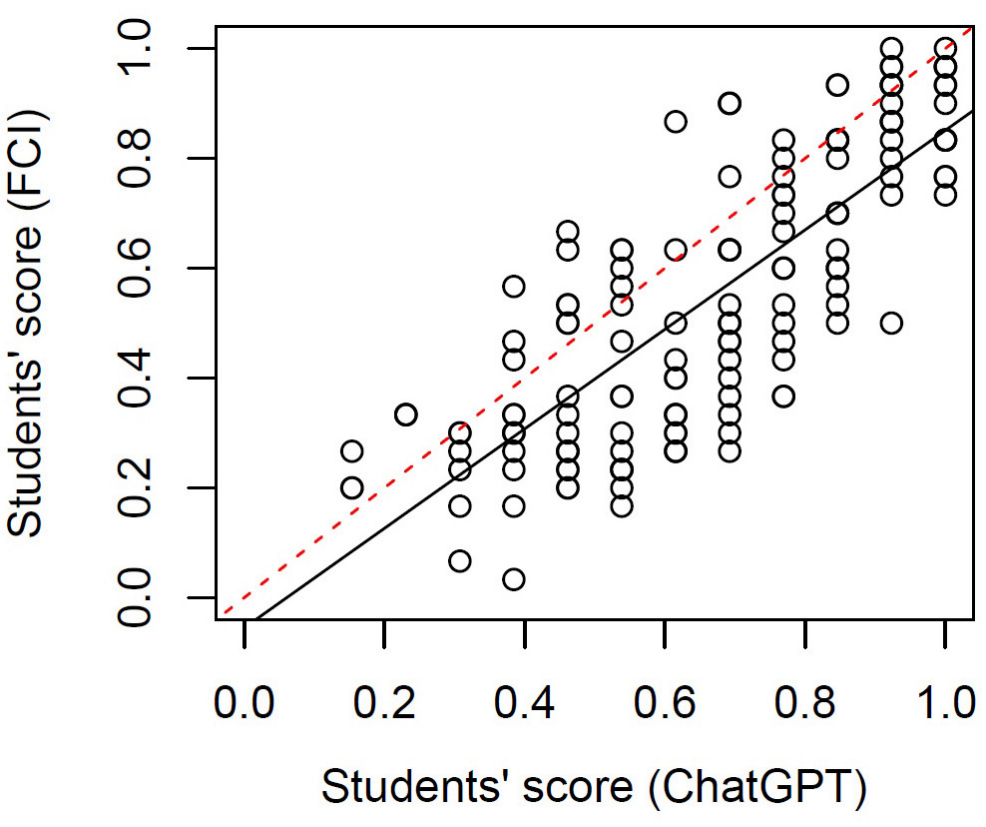
Students' score in FCI items vs. their score in ChatGPT items. The black solid line represents a linear regression between the two variables and the red dashed line is a guide to the eye with a slope of 1.

We analyzed the performance in the ChatGPT items and the FCI to understand how closely related the two sets of items are and how valid they are to map the concepts that are assessed in the FCI. Figure 4 shows the students' scores in both item sets. We found a significant linear regression with a slope of 0.70 (*p* < 10^−10^), Pearson's correlation coefficient *r* = 0.79), i.e., there is a very strong relation between the two set of items. Moreover, it is noticeable that over the whole range of students' scores, their performance in ChatGPT items is higher than their performance in FCI items. This means that for low, medium and high performers, ChatGPT items were easier than the FCI items.

### 4.3 Confirmatory factor analysis

#### 4.3.1 Factor structure of the FCI

Prior research has suggested three different factor structures for the FCI (Eaton and Willoughby, 2018). Here, we found that only the model suggested by Eaton and Willoughby (2018) (E&W) adequately describes the FCI data. The factor model described by Scott et al. (2012) and the originally intended factor structure by Hestenes et al. (1992) did not converge, i.e., both factor models do not describe the data properly. After testing the original model by E&W, we moved item 16 of the FCI as part of the factor Newton 1 + Kinematics in the model by E&W, because item 16 addresses the force on a truck that is pushed by a car with a constant speed. Here, two concepts are relevant: Newton's first law and the relation between velocity and acceleration. Placing this item into 1st law+kinematics led to an improved fit to the data and the final model (E&W mod) that describes best the data is shown in Table 1. Table 2 shows the results of the confirmatory factor analysis of this model. It is noticeable that the model exhibits acceptable fit statistics with the added residual correlations [CFI > 0.9, TLI > 0.9, SRMR < 0.08, RMSEA (Upper Cl) < 0.06].

**Table 1:** The factor model suggested by Eaton and Willoughby (2018) with the modification that item 16 is part of the factor “1st Law + Kin.”

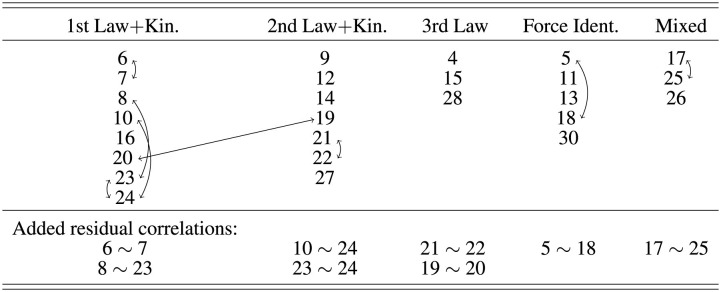

The numbers in the table indicate the item number. The added residual correlations are based on expert considerations (Eaton and Willoughby, 2018; Scott et al., 2012), and they are indicated by the arrows.

**Table 2:** Summaries of the confirmatory factor analyses.

**Model**	**Items**	**No. of factors**	**No. of Res. Cor**.	**CFI**	**TLI**	**SRMR**	**RMSEA**	**AIC**	**BIC**
E&W mod	FCI	5	8	0.92	0.91	0.06	0.04	5,491	5,718
GPT HWS	GPT	5	0	0.88	0.85	0.06	0.05	3,022	3,148
GPT EW	GPT	3	0	0.90	0.88	0.07	0.04	2,835	2,933
GPT EW	GPT	3	1	0.95	0.94	0.06	0.03	2,825	2,926
GPT EW + E&W	FCI + GPT	5	9	0.88	0.87	0.07	0.04	8,103	8,425

The statistics include the Comparative Fit Index (CFI), the Tucker-Lewis Index (TLI), the Standardized Root Mean Square Residual (SRMR), and measures for the parsimony of the fit, namely the Root Mean Square Error of Approximation (RMSEA), the Akaike information criterion (AIC), and the Bayesian information criterion (BIC).

#### 4.3.2 Factor structure of GPT items

Regarding the factor structure of the GPT items, we considered an analog structure to the two expert models reported for the FCI, i.e., the model by Hestenes et al. (1992) and the one by Eaton and Willoughby (2018). We did not consider an analog model to the factor model found by Scott et al. (2012) in an exploratory factor analysis here, because this model was purely data-driven and it is difficult to translate to GPT items.

The factor model by Hestenes et al. (1992) considers six factors, and the items have been designed accordingly to match these factors (Eaton and Willoughby, 2018). In our selection of concepts for the GPT items, we also followed five of these factors, namely kinematics, Newton's 1st law, Newton's 2nd law, Newton's 3rd law, and superposition. In this paper, we call this model “GPT HWS,” referring to the first letters of the three authors of the corresponding FCI factor model, Hestenes, Wells, and Swackhamer. Each factor in the GPT HWS model contains three items as it was intended in the item design process without residual strains. Table 2 shows that this model does not reach an acceptable range of fit statistics (CFI < 0.9, TLI < 0.9).

As mentioned above, the factor model by Eaton et al. includes five factors: Newton's 1st law and kinematics, Newton's 2nd law and kinematics, Newton's 3rd law, force identification, and mixed. These factors account for the fact that it is sometimes useful to assess the understanding of Newton's first and second law in a kinematics context and they do not separate between these two concepts within an item. The GPT items created here can be well mapped on these categories. Table 3 shows the factor structure of the GPT model that is aligned with the E&W model. Here, the items that target Newton's first law are not related to kinematics and therefore the first factor is renamed to Newton's first law. The items that target superposition and kinematics are closer related to the second law and therefore they are both included in the second factor. Naturally, the items that assess Newton's third law form an independent third factor as in the E&W model.

**Table 3:** The factor model of ChatGPT items suggested in this work.

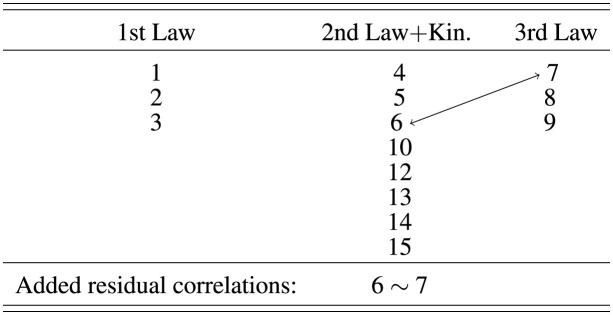

The numbers in the table indicate the item number. The added residual correlations are based on expert considerations.

We tested this model without internal strains and found that the fit statistics are at the acceptable limit of CFI = 0.90 and TLI = 0.88. When considering a plausible residual correlation between GPT items 6 and 7, it leads to very good model fit of CFI = 0.95 and TLI = 0.94. Also this model has the higher parsimony with RMSEA = 0.03, AIC = 2,825 and BIC = 2,926. Therefore, this model fits the GPT items best (see Table 3).

We also created a joint model of FCI and GPT items in which we merge the best models for the two individual item sets (termed GPT EW + E&W in Table 2). This means, we combined the modified model by Eaton and Willoughby (2018) for the FCI data with five factors and eight residual correlations and the best model for GPT items that is aligned with the model for the FCI items by Eaton and Willoughby (2018), which has three factors and one residual correlation. Since the factors are already aligned, the combined model consists of five factors and nine residual correlations. The created model represents the best combination of the two item sets, but the fit statistics are below the acceptable range (CFI < 0.9, TLI < 0.9).

### 4.4 Exploratory factor analysis

The purpose of the exploratory factor analysis (EFA) was to investigate whether ChatGPT items load on the same factors as the FCI items. This would mean that the items created by ChatGPT and FCI items require a comparable underlying conceptual understanding to solve them.

We verified the equality of variances of the samples, which is one of the conditions for the EFA, using Bartlett's test (*p* < 10^−150^) and the sampling adequacy for each item using the Kaiser-Meyer-Olkin (KMO) test. The KMO Test resulted in an overall measure of sample adequacy (MSA) of 0.84 with a minimum of 0.58 (above the limit of 0.5) in one of the ChatGPT items. This means that the data is suitable to perform an EFA.

To determine the number of factors optimally suited to describe the data, we created a Scree plot and performed a parallel analysis. Both analyses suggested an optimal number of three factors. A chi square hypothesis test confirmed that three factors are sufficient to describe the data (*p* < 10^−4^). The factor loadings of the exploratory factor analysis with three factors using Varimax rotation are shown in Table 4 in the Supplementary material.

Overall, the results of the factor analysis indicate that both sets of items, ChatGPT and FCI items, load on the same factors. In both sets, those items that were associated with the factor “Newton's 1st law + kinematics” during the CFA mainly load on factor 2 of the EFA. Items that are part of the factor “Newton's 2nd law + kinematics” (CFA) load on factors 1 and 2 (EFA), and items of the factor “Newton's 3rd law” (CFA) load on factor 3 (EFA).

## 5 Discussion

### 5.1 Content validity rated by experts

Our results show that physics education experts rated the quality of items created with ChatGPT after careful prompt engineering and significant exclusion of 70 out of 100 items with high scores in four categories. This means that ChatGPT is able to embed the item in a meaningful context even though previous research shows that prospective physics teachers often fail to do so (Küchemann et al., 2023). Moreover, the items exhibited a high clarity and appropriate difficulty level, which is in line to observations by earlier research (Küchemann et al., 2023). It is interesting that the items also had a high specificity, i.e., all relevant information to solve the items were given. Previous research observed that both physics items created by prospective physics teachers who used a textbook and those who used ChatGPT to create them lacked a high specificity (Küchemann et al., 2023). Only the concept of superposition did not reach highest expert ratings (see Supplementary material). Here, the item lacked specificity, clarity, and were partially misleading. The distractors were also ambiguous, and the concept items were also rather similar. This observation shows that LLMs may have difficulties creating high-quality items for certain concepts.

mainrowheight16.5pt

In practice, it is unlikely that educators can invest a similar effort to create a large amount of items, exclude about 70% of them, and then perform an expert rating with peers to obtain an optimal set of items. Instead, it is more likely and time-efficient to manually optimize a set of items created by ChatGPT. In this work, we did not investigate the influence of the details of the prompt or the categories of the expert ratings on the psychometric quality of the items. Therefore, our results do not allow us to conclude on an optimal sequence of steps that educators need to follow to obtain an optimal set of items. However, we found frequent errors in the items generated by ChatGPT, which are known to affect the psychometric quality of the items and subsequently led to their exclusion (step 3 in Section 3.2) (Moreno et al., 2006; Raina and Gales, 2022). Thus, in consideration of previous works (Moreno et al., 2006; Raina and Gales, 2022; Küchemann et al., 2023) and the observations in this work, we suggest that the manual optimization process considers the following aspects:

1. the physical correctness of the item stem and the answer alternatives in case of multiple-choice items (this work),
2. the inclusion of the main point in the item statement (Moreno et al., 2006),
3. the difficulty of the item is appropriate for the target group (Moreno et al., 2006),
4. the distinction of each item to other generated items (this work),
5. the fulfillment of the format requirements mentioned in the prompt, such as the correctness of exactly one answer alternative (Moreno et al., 2006 and this work),
6. the phrasing of the item that it does not contain or suggest the correct answer (Moreno et al., 2006 and this work),
7. the inclusion of all relevant information in the item stem (Küchemann et al., 2023),
8. the fact that the item stem and (in case of multiple-choice items) the answer alternatives are phrased clearly and not misleading (Raina and Gales, 2022), and
9. that the answer alternatives of a multiple-choice item are neither ambiguous nor very similar to each other (Moreno et al., 2006).

We believe that these suggestions may support the manual optimization process.

### 5.2 Characteristics of GPT items

In this work, we created the GPT items to assess the understanding of five concepts that are also part of the FCI. Then, we compared the results of the CFA of ChatGPT items to the ones of FCI items because the CFA reveals the underlying factor model that describes the data best. The intention of the developers of the FCI was to assess the understanding of specific concepts, namely superposition, Newton's first law, Newton's second law, Newton's third law and kinematics. To objectively measure students' conceptual understanding, it is necessary to develop sets of items in a way that each set only requires the understanding of a single concept (unidimensionality, see for instance Wright, 1997; Planinic et al., 2019). This means that in an ideal case, the factor model would have five factors, where each factor consists of items that separately assess one of these concepts. In fact, Eaton and Willoughby demonstrate that the factor model originally intended by the developers does describe students' responses to the FCI items (Eaton and Willoughby, 2018). Contrary to this previous result (see also Section 2.1), we found that a model that is based on our originally intended factor structure with five factors cannot describe the data. Instead, we found that the factor structure of GPT items can be fit by another expert model previously reported by Eaton and Willoughby (2018). This fit model also consists of five factors, namely Newton's 1st law and kinematics, Newton's 2nd law and kinematics, and Newton's 3rd law, force identification and mixed. Analog to this model, the model used here to describe the GPT items consist of three factors namely Newton's 1st law, Newton's 2nd law and kinematics, and Newton's 3rd law. The items that have been previously designed to assess the concepts of superposition and kinematics belong to the second factor (Newton's 2nd law and kinematics). This also means that the item sets created by ChatGPT were unable to assess some concepts, such as superposition and kinematics, independently from Newton's 2nd law. For concept inventories, a one-dimensional structure exhibits a high relevance to assess the students' isolated understanding of specific concepts and not only in a relation to other concepts (Wright, 1997; Planinic et al., 2019). Consequently, similar to the FCI items, ChatGPT also does not create sets of unidimensional items. Therefore, the objective diagnosis of students' understanding and difficulties using ChatGPT items would be compromised. At this stage, it is unclear whether state-of-the-art LLMs would be able to design items assessing superposition or kinematics without the need of other concepts if they are specifically prompted to exclude these concepts in the items. If not, human revision would be indispensable.

Moreover, we found that the GPT items were overall easier for students, they had a lower discrimination index, and lower point biserial index. One reason for this may be that the distractors were not aligned with misconceptions and therefore not as strong as in typical concept items. As mentioned above, distractors in concept tests are usually created after evaluating students open responses to the items. In this way, the distractors are closely aligned with students' conceptions. Therefore, our findings underscore the importance of including students' open responses or expert opinions who have experiences with students' difficulties in the concept inventory design process.

In general, we think that the quality of the output of large language models for education purposes is difficult to determine. There are benchmark tests that allow the qualification of performance regarding specific tasks, but none of them are specifically related to education (Touvron et al., 2023). Therefore, we decided to choose a task that is very common in education (namely item creation), and where the performance in this task can be objectively determined. Due to the high effort in empirical data collection, we invested a lot of time in the selection and analysis of the items. Therefore, we wanted to optimize the outputs in the best possible way beforehand. This allowed us to obtain an upper performance limit that can be reached without manual correction and determine the performance of LLMs in a common task in education similar to standard benchmark tests.

### 5.3 Limitations

In this work, we used a specific large language model, namely ChatGPT 3.5, it is not the most recent version that reaches the highest performances in benchmark tests and allows a multimodal input and output. In general, we need to restrict our findings to ChatGPT 3.5 and we cannot say which large language model would perform better or worse in creating physics items in mechanics. We do not have information on the model size or training data set of ChatGPT 3.5, and therefore, we cannot say what is the required size or number and type of training instances to achieve the findings we obtained here. We can assume that newer models, such as ChatGPT 4 and Gemini, that reach higher performances in benchmark tests have more parameters and have been trained on a larger data set. Therefore, it is likely that our findings are transferable and may be even exceeded by these newer models that are likely to have both, more parameters and a larger training data set.

Furthermore, there were only 173 students who took part in the study. Even though there is no specific size criteria for a confirmatory factor analysis, it is important to have a large number of students who take part in it to reveal an underlying factor structure. Even though we found a factor structure that describes the data well, we cannot exclude that a larger number of students may yield in a satisfactory fit of another factor model.

In this line, a large part of the target group were undergraduate STEM students who may or may not have received an instruction on the mechanics concepts immediately prior to participation. However, the physics curricula of high schools and/or middle schools of every state in Germany cover the concepts of motion and Newton's mechanics, and they are a part of Disciplinary Core Ideas in Wisconsin's Standards for Science. Moreover, understanding motion and forces is also part of the National Science Education Standards and part of the Next Generation Science Standards. Therefore, it is reasonable to assume that the participants have studied the concepts at some point prior to participation. Nonetheless, it is known that it is more difficult to reveal a factor structure when students often guess answers. To account for this effect, we identified a temporal cutoff and removed the students who spent a shorter time on the entire questionnaire then the time of the cutoff. In this way, we intended to remove the students who guessed several answers. However, we cannot exclude that there were still students in the final sample who guessed the answers.

Apart from that, we tested the capacity of ChatGPT 3.5 to create concept items in a topic that is one of the most common topics in physics. Therefore, it is likely that these topics are part of the training data set. But, at this point, we do not know how ChatGPT 3.5 or at the language models would perform in other fields or in topics that are less common.

## 6 Conclusion

In sum, we can conclude that large language models in general and ChatGPT 3.5 exhibit the capacity to create concept items. Even though we tested a specific language model, we can assume that newer models, such as ChatGPT 4 and Gemini, are able to perform similarly or even exceed the quality of physics items obtained in this work. Nevertheless, based on the findings of this work, it is a plausible conjecture that it is necessary to manually align distractors with students' thinking and difficulties to increase overall difficulty levels of items. It is also important to consider that the distractors created for the physics items by ChatGPT are not aligned with students' difficulties. This means that the concept items created by ChatGPT cannot be considered to have a diagnostic capacity, and it is necessary that experts revise the distractors to be able to detect students' difficulties more accurately. Moreover, it is necessary to consider that large language models may not be able to create high-quality items for all concepts and human review is also necessary in this aspect.

In future research, it would be helpful to study how a training data set needs to be designed to enhance the conceptual understanding and ability to create concept items of large language models. Ideally, the distractors in automatically created concept items would be well aligned with students' difficulties and that the created items by a large language models exhibit some diagnostic capacity. Moreover, it would be helpful to investigate how to create concept items with a large language model that would exhibit a single dimension and do not accept more than one contact at a time.

## Data Availability

The original contributions presented in the study are included in the article/Supplementary material, further inquiries can be directed to the corresponding author.
