# Supplementary material for: ChatGPT's quality: Reliability and validity of concept inventory items
Source: Front Psychol. 2024 Oct 8;15:1426209. doi: 10.3389/fpsyg.2024.1426209 (PMC11493723; doi:10.3389/fpsyg.2024.1426209)
Supplement: Supplementary file 1 [file Data_Sheet_1.PDF]

Quality criteria

Item stem

Specificity: This criterion indicates whether the item is specific, i.e. whether all relevant information is given to solve the task. If this is the case, a 1 is assigned; if it is not clearly formulated, a 0 is given.

Clarity: This criterion indicates whether the item is formulated clearly and concisely. If this is the case, a 1 is awarded; if it is too lengthy or cumbersome, a 0 is given.

Correctness: This criterion indicates whether the item is formulated correctly. If this is the case, a 1 is awarded; if there is an error in the task formulation, a 0 is given.

Not misleading: This criterion indicates whether the item is not misleading or misleading. If this is the case, a 1 is awarded; if not, a 0 is given.

Answer alternatives

Not ambiguous: This criterion is used to indicate whether the answer options are unambiguous. If this is the case, a 1 is awarded; if it is not unambiguous, a 0 is awarded.

Similarity: This criterion should indicate whether the incorrect answer options are clearly distinguishable from the correct answer. If this is the case, a 1 is awarded; if it is not clearly formulated, a 0 is awarded.

Not misleading: This criterion is intended to indicate whether the answer options are not misleading or misleading, so that one could tend to get a false picture of the concept or situation. If it is not misleading, 1 point is awarded; if it is not clearly formulated, a 0 is awarded.

One correct answer and four incorrect options: This criterion indicates whether there are exactly four incorrect and one correct answer options so that the format is consistent across all questions. If this is the case, 1 point is given; if it is not clearly formulated, a 0 is awarded.

Entire item

Fluency: This criterion should indicate whether the question and the answer options are grammatically consistent in their wording. Do the answer options represent a grammatically correct answer to the question? If this is the case, a 1 is awarded; if it is not clearly formulated, a 0 is awarded.

Relevance: This criterion should indicate whether the concept is relevant for answering the question, i.e. are partial aspects of the concept tested in the task. It is not yet a question of whether other concepts are also relevant to the answer. If this is the case, a 1 is awarded; if it is not clearly formulated, a 0 is awarded.

Appropriate difficulty: This criterion indicates whether the question has an appropriate level of difficulty for upper secondary and undergraduate students. If this is the case, a 1 will be awarded; if it is not clearly formulated, a 0 will be awarded.

1 Dim: It means that no understanding other than that of the target concept is required. This criterion indicates whether another concept than the one, the items targets, is relevant. This is to ensure in advance that mine has a clear factorial structure. If this is the case, a 1 is awarded; if it is not clearly formulated, a 0 is awarded.

Cognitive task: This criterion indicates whether the item requires a cognitive activity related to Bloom's taxonomy levels "apply" or "evaluate". If this is the case, a 1 will be awarded; if it is not the case, 0 points will be awarded.

Diversity: This criterion indicates whether the item is not too similar to other items in the test. If this is the case, 1 point will be given; if it is too similar, a 0 will be awarded.

Context: This criterion indicates whether the item is embedded in an appropriate context. If this is the case, 1 point will be awarded; if it is not clearly formulated, a 0 will be awarded.

Expert rating according to quality criteria

Item	Item stem				Answer Alternatives				Entire item							Final Score
	Speci-ficity	Clarity	Correct-ness	Not Mis-leading	Not ambi-guous	Similarity	Not mis-leading	One correct and four incorrect answers	Fluency	Rele-vance	Appro-priate Difficulty	1 Dim	Cognitive task	Diversity	Context	
**Newton's 1st Law**																
1	1	1	1	1	1	1	1	1	1	1	1	1	1	1	1	1,00
2	1	1	1	1	1	1	1	1	1	1	1	1	1	1	1	1,00
3	1	1	1	1	1	1	0	1	1	1	1	1	1	1	1	0,93
**Newton's 2nd Law**																
4	1	1	1	1	1	1	1	1	1	1	1	1	1	1	1	1,00
5	1	1	1	1	1	1	1	1	1	1	1	1	1	1	1	1,00
6	1	1	1	1	1	1	1	1	1	1	1	1	1	1	1	1,00
**Newton's 3rd Law**																
7	1	1	1	1	1	1	1	1	1	1	1	1	1	1	1	1,00
8	1	1	1	1	1	1	1	1	1	1	1	1	1	1	1	1,00
9	1	1	1	1	1	1	0	1	1	1	1	1	1	1	1	0,93
**Superposition**																
10	0	0	1	0	1	1	1	1	1	1	1	1	1	0	1	0,73
11	0	1	1	0	0	1	0	1	1	1	1	1	1	0	1	0,67
12	1	1	1	1	0	1	0	1	0	1	1	1	1	1	1	0,80
**Kinematics**																
13	1	1	1	1	1	1	1	1	1	1	1	1	1	1	1	1,00
14	1	1	1	1	1	1	1	1	1	1	1	1	1	1	1	1,00
15	1	1	1	1	1	1	0	1	1	1	1	1	1	1	1	0,93

Items created by ChatGPT

1) A person is standing inside an elevator that is moving upwards at a constant speed. What is the person experiencing?

A. The person is being pulled down by a gravitational force.

B. The person is being pushed up by air resistance.

C. The person is accelerating at a constant speed.

D. The gravitational pull from the Earth cancels out with the normal force from the floor.

E. The person is floating in a vacuum.

2) Consider a scenario where you are playing catch with a friend and the ball suddenly slips out of your hand. What would happen to the velocity of the ball as it falls to the ground after slipping out of your hand?

A. It would decrease as it falls.

B. It would remain the same.

C. It would increase as it falls.

D. It would alternate between increasing and decreasing.

E. It would first increase and then decrease.

3) Imagine you are inside a space shuttle in orbit around the Earth. You are feeling weightless. What can be said about the motion of the space shuttle?

A. It moves at a constant speed in a straight line.

B. It moves at an increasing speed in a straight line.

C. It moves at a decreasing speed in a straight line.

D. It moves at a constant speed in a curved line.

E. It moves at an increasing speed in a curved line.

4) A soccer ball is kicked with a certain force. How does the ball's momentum change as a result?

a) It decreases

b) It increases

c) It remains the same

d) It first increases and then decreases

e) It first decreases and then increases

5) A car is traveling at a constant speed, but then comes to a stop. What happens to the car's momentum as a result?

a) It decreases

b) It increases

c) It remains the same

d) It first increases and then decreases

e) It first decreases and then increases

6) A ball is dropped from a high tower and its velocity changes as it falls due to the force of gravity. What is the relationship between the net force acting on the ball and its acceleration?

A) The net force is equal to the acceleration

B) The net force is proportional to the acceleration

C) The net force is inversely proportional to the acceleration

D) The net force is proportional to the square of the acceleration

E) The net force is proportional to the square root of the acceleration

7) A child is playing on a playground and is pushing a friend on a swing. The friend is swinging higher and higher. Who is applying more force on the other person?

A) The child is applying more force

B) The friend is applying more force

C) They are both applying equal force

D) The push force is proportional to the speed of the swing

E) The push force is proportional to the height of the swing

8) In a frictionless hockey rink, a player hits a puck with a stick. The puck travels at a constant speed of 20 m/s. What can be said about the force exerted by the stick on the puck and the force exerted by the puck on the stick?

A) The force exerted by the stick on the puck is greater than the force exerted by the puck on the stick

B) The force exerted by the puck on the stick is greater than the force exerted by the stick on the puck

C) The force exerted by the stick on the puck is equal in magnitude and opposite in direction to the force exerted by the puck on the stick

D) The force exerted by the stick on the puck is equal in magnitude but not opposite in direction to the force exerted by the puck on the stick

E) The force exerted by the puck on the stick is equal in both magnitude and direction to the force exerted by the stick on the puck

9) While playing catch with a friend, you throw a ball with a force of 30 N. Your friend catches the ball. What can be said about the force exerted by the ball on your friend's hand and the force exerted by your friend's hand on the ball?

A) The force exerted by the ball on your friend's hand is greater than the force exerted by your friend's hand on the ball.

B) The force exerted by your friend's hand on the ball is greater than the force exerted by the ball on your friend's hand.

C) The force exerted by the ball on your friend's hand is equal in magnitude but not opposite in direction to the force exerted by your friend's hand on the ball.

D) The force exerted by the ball on your friend's hand is equal in magnitude and opposite in direction to the force exerted by your friend's hand on the ball.

E) The force exerted by the ball on your friend's hand is equal in both magnitude and direction to the force exerted by your friend's hand on the ball.

10) You are playing catch with a friend and you throw the ball diagonally at a 45 degree angle. The ball reaches the peak of its trajectory and starts to fall back down. Which component of velocity affects the time it takes for the ball to reach the peak?

a) Horizontal component

b) Vertical component

c) Both components equally

d) Neither component

e) The ball reaches the peak instantly

11) When jumping off a diving board, which of the following best describes the relationship between the horizontal and vertical velocity components?

A) The horizontal velocity component is always equal to the vertical velocity component.

B) The horizontal velocity component is always greater than the vertical velocity component.

C) The horizontal velocity component is always less than the vertical velocity component.

D) The horizontal and vertical velocity components are independent and can have any relationship.

E) The horizontal velocity component is proportional to the vertical velocity component.

12) A basketball player throws a ball diagonally to the basket, what type of motion does the ball have in the vertical direction?

A) Simple Harmonic

B) Circular

C) Constant

D) Accelerated

E) Linear

13) A ball is thrown vertically upwards and reaches a height of 30 meters after 5 seconds. What can be concluded about the ball's acceleration?

A) The ball's acceleration remained constant throughout the 5 seconds.

B) The ball's acceleration increased throughout the 5 seconds.

C) The ball's acceleration decreased throughout the 5 seconds, but at a constant rate.

D) The ball's acceleration decreased throughout the 5 seconds, but at an increasing rate.

E) The ball's acceleration decreased throughout the 5 seconds, but at a decreasing rate.

14) You are riding in a car that starts from rest and takes 5 seconds to reach a speed of 60 mph. What is the acceleration of the car?

A. 12 mph/s

B. 8 mph/s

C. 10 mph/s

D. 6 mph/s

E. 7 mph/s

15) A car is driving down the highway at a constant acceleration. What happens to the car's velocity?

a) The velocity decreases

b) The velocity stays the same

c) The velocity increases in a linear manner

d) The velocity increases, but at a decreasing rate

e) The velocity increases, but at an increasing rate

Table 4. Factor loadings of the EFA. Loadings below a threshold of 0.16 and loadings with a difference larger than 0.1 below the maximum loading for each item were excluded for clarity.

Item	Factor 1	Factor 2	Factor 3
ChatGPT 1	0.3		
ChatGPT 2		0.33	
ChatGPT 3		0.32	
ChatGPT 4	0.28	0.28	0.23
ChatGPT 5	0.21	0.26	0.20
ChatGPT 6	0.34	0.30	
ChatGPT 7			0.65
ChatGPT 8			0.64
ChatGPT 9		0.37	0.40
ChatGPT 10	0.29	0,36	
ChatGPT 11		0.43	
ChatGPT 12		0.22	
ChatGPT 13	0.48	0.28	
ChatGPT 14		0.36	
ChatGPT 15		0.45	
FCI 1		0.36	
FCI 2	0.34		0.31
FCI 3	0.31	0.36	
FCI 4			0.65
FCI 5	0.59		
FCI 6		0.38	0.32
FCI 7		0.38	
FCI 8	0.40		
FCI 9	0.48		
FCI 10		0.60	
FCI 11	0.47		
FCI 12		0.39	
FCI 13	0.62		
FCI 14	0.30	0.31	
FCI 15			0.60
FCI 16		0.48	
FCI 17	0.66		
FCI 18	0.57		
FCI 19		0.43	
FCI 20		0.49	
FCI 21	0.40		
FCI 22	0.39		
FCI 23	0.38	0.45	
FCI 24		0.54	
FCI 25	0.66		
FCI 26	0.49		
FCI 27		0.43	
FCI 28			0.61
FCI 29			0.16
FCI 30	0.46		
